# Pediatric acute liver failure: An experience of a pediatric intensive care unit from resource limited settings

**DOI:** 10.3389/fped.2022.956699

**Published:** 2022-09-02

**Authors:** Puja Amatya, Sudeep Kumar Kapalavai, Akash Deep, Srinivas Sankaranarayanan, Ravikumar Krupanandan, Kalaimaran Sadasivam, Bala Ramachandran

**Affiliations:** ^1^Department of Pediatric Critical Care, Kanchi Kamakoti CHILDS Trust Hospital, Chennai, India; ^2^Department of Pediatric Critical Care, King's College Hospital, London, United Kingdom; ^3^Department of Gastroenterology, Kanchi Kamakoti CHILDS Trust Hospital, Chennai, India

**Keywords:** pediatric acute liver failure, acute liver failure, King's College Hospital Criteria, international normalized ratio, liver transplantation, spontaneous regeneration

## Abstract

**Introduction:**

Pediatric acute liver failure is a rare and serious disease. Though liver transplantation is considered as the established treatment option for patients who are unlikely to recover with medical management, however, with the advancement of medical care there has been an increase in spontaneous regeneration of liver, obviating the need for liver transplantation. We identified the etiologies, outcome and prognostic factors of acute liver failure and the validity of the existing liver transplantation criteria to predict the outcome of pediatric acute liver failure.

**Materials and methods:**

This was a retrospective study done from January 2014 to December 2019 in a tertiary pediatric critical care unit in South India. All children aged between 1 month to 18 years admitted with acute liver failure were enrolled.

**Results:**

Of 125 children with acute liver failure, the main etiologies were infections (32%), indeterminate (23%), paracetamol toxicity (21%), metabolic (13%) and others (11%). Dengue was the most common infection (55%). The median pediatric logistic organ dysfunction score at admission was 12 (4–27). Of 125 patients, 63.2% (*n* = 79) had spontaneous regeneration which was higher in paracetamol induced (92.3%) compared to non-paracetamol induced acute liver failure (55.5%). Only two patients underwent liver transplantation and 35% died. Peak alanine transaminase and use of inotropes significantly predicted the outcome of disease. Of 38 children meeting King's College Hospital criteria for liver transplantation, 57.9% had spontaneous regeneration and 36.8% died. Of 74 children meeting INR > 4 criteria, 54% (*n* = 40) had spontaneous regeneration and 43.2% died. INR >4 criteria was more sensitive than King's College Hospital criteria for predicting the need for liver transplantation.

**Conclusion:**

Pediatric acute liver failure is caused by varied etiologies and infections were the commonest cause. Despite having a seriously ill cohort of patients, medical management resulted in spontaneous regeneration in the majority of children with acute liver failure. The use of inotropes, advanced hepatic encephalopathy, and peak alanine transaminase were predictors of poor outcome in children with acute liver failure and these patients could be considered for liver transplantation as available. Therefore, we may need to develop better predictors of pediatric acute liver failure in resource limited settings.

## Introduction

Pediatric acute liver failure (PALF) is a potentially fatal condition caused by various etiologies. The PALF study group recommended PALF definition as follows: (a) evidence of liver dysfunction within 8 weeks of onset of symptoms, (b) uncorrectable (6–8 h after administration of one dose of parenteral vitamin K) coagulopathy with international normalized ratio (INR) >1.5 in patients with hepatic encephalopathy (HE) or INR> 2.0 in patients without HE, and (c) no evidence of chronic liver disease either at presentation or in the past (1). The etiology of acute liver failure (ALF) varies according to the age of the patient, geographical location and socio-economic status of the country (2–4). PALF is caused mainly by infectious etiologies in developing countries whereas indeterminate cause is most common in developed countries in Europe and North America (5). Similarly, the etiology of ALF in the United States in adults is mainly due to drugs, notably paracetamol (PCM), and hepatotropic viruses, whereas in children it is mainly due to indeterminate etiologies (6–8).

Emergency liver transplantation (LT) remains the only definitive treatment for those patients with PALF who are unlikely to recover with intensive medical treatment, PALF accounts for 10–15% of all pediatric LTs (9, 10). Survival with spontaneous regeneration (SR) of the liver with medical therapy alone is increasing. This is probably due to improvements in our understanding of the underlying pathophysiology of PALF and critical care management of patients with ALF. King's College Hospital, London (11) reported spontaneous regeneration (SR) in 28% and death in 72% of patients with ALF in the pre-transplantation era. After LT era, the PALF Study database demonstrated a much higher SR of 56% (1999–2004). The Pediatric Health Information System, from the United States (12) also reported an increase in SR to 73.4% (2008–2012). Outcome of ALF with SR varies according to the etiology, with survival being better in PCM overdose and worse in children with indeterminate etiology (4, 13).

Though several scoring systems are available to predict mortality in non-transplanted ALF patients, optimal prognostic criteria for poor outcome without LT are still lacking (14). The King's College Hospital Criteria (KCHC), formulated in 1989, are the most extensively studied and widely used in ALF patients (15). As PALF is different from adult ALF in various aspects like definition, presentation, etiologies and outcome (13, 16, 17), the KCHC may not be applicable to assess prognosis in PALF.

In addition, there is a paucity of information regarding etiology, prognostic factors for PALF, and the applicability of KCHC in Indian settings. There are differences in resources, organ allocation and kind of LT between high and lower middle-income countries (LMICs). By applying criteria developed for a different population to patients with PALF, it is possible that some children who may recover spontaneously are listed for LT and some children who were thought to be spontaneously recovering might require LT or die before LT. This is extremely important as not all centers have well-established LT programmes especially due to financial and logistical reasons. Therefore, we conducted this study to identify the etiologies, outcomes, prognostic factors for survival with SR and the validity of LT criteria in predicting SR in children with PALF with limited access to LT.

## Materials and methods

This was a retrospective study done at a tertiary care children's hospital in South India. This is a 200 bedded children hospital with an annual admission of 12,400 patients, 14 Pediatric Intensive Care Unit (PICU) beds and 7 high dependency (HDU) beds, with approximately 800 PICU admissions annually. This center provides tertiary level care to seriously ill children from different parts of South India. Most of our patient population comes from Low- and Middle-Income socio-economic groups and do not have medical insurance. All children with PALF are admitted to the PICU or HDU, based on the clinical condition. All children aged 1 month to 18 years admitted to the PICU who met the criteria of PALF as defined by the PALF study group definition (1) at admission or over a period of hospitalization were included. Neonates and children with chronic liver disease or failure were excluded. Data were collected from the PICU electronic database and hospital medical records. All the children with PALF were managed according to a pre-defined protocol. Patients with HE Grade I and II were managed with supportive treatment, including maintaining euglycemia, monitoring electrolytes and ammonia, bowel regimens (lactulose), close observation of sensorium, transfusion of blood products for active bleeding, and use of appropriate antimicrobials. Patients with progressive HE or > grade III HE were managed by intubation, neuroprotection and neurological imaging studies, in addition to the interventions mentioned above. Prophylaxis for gut protection was added in children who were kept nil by mouth. Extracorporeal measures like renal replacement therapy (RRT) and plasmapheresis were performed based on serum ammonia level, grade of encephalopathy, presence of acute kidney injury (AKI), hyperlactatemia, acid-base imbalance and presence of hyperbilirubinemia. Criteria for LT were based on our unit protocol which included KCHC (14) and UK organ allocation based on INR > 4 (18). Since LT was not available in our center, all children who fit the criteria for LT were counseled and referred to a center where LT was available, if the family agreed. Those patients who did not opt for transfer to a LT center were managed medically. Data regarding demographic profile, clinical features, investigations and treatment were collected. The etiologies of PALF were divided into five groups – Indeterminate, Paracetamol toxicity, Infections, Metabolic and Others. The category ‘Others' included drug induced liver failure excluding paracetamol, acquired immunodeficiency syndrome, poisoning and multi-organ dysfunction syndrome (MODS). The outcome of PALF was divided into two groups: spontaneous regeneration (SR) and non-spontaneous regeneration [NSR – (LT or death)]. Bleeding symptoms were divided into two categories - major and minor. In major bleeding, gastrointestinal, pulmonary and intracranial hemorrhage were included whereas skin and mucosal bleeding were classified as minor. Liver Injury Units (LIU) Scoring System was used to calculate LIU score. LIU score was calculated by the following formula. LIU = [3.584 × peak total bilirubin (mg/dL)] + [1.809 peak prothrombin time (PT) (seconds)] + [0.307 × peak ammonia (micro mol/L)]. Alternatively, substituting international normalized ratio (INR) for PT, the score was calculated as LIU = (3.507 × peak total bilirubin) + (45.51 × peak INR) + (0.254 × peak ammonia) substituting INR for prothrombin time (19).

### Ethical approval

The study was approved by the hospital Institutional Review Board (IRB) - approval number KKCTH-CTMRF: IEC-DNB/37/March 2019 (IRB min. dt. 07.03.2019).

### Statistical analysis

Data were analyzed using SPSS 21.0 software (IBM). In univariate analysis, the qualitative data were analyzed with Pearson's chi-square test and quantitative data were analyzed using the student *t*-test and Mann-Whitney *U* test. The significant variables were then analyzed with multivariate logistic regression analysis. We defined sensitivity, specificity, positive predictive value (PPV) and negative predictive values (NPV) for KCHC and INR >4 in predicting outcomes for both SR and NSR group. The optimal cut-off points predicting the outcome of patients with ALF for total serum bilirubin, alanine transaminase (ALT), ammonia, INR, lactate, pH, albumin, lowest sodium, PELOD, and Liver Injury Units (LIU) score were created using receiver operating characteristic (ROC) curve using Younden index.

## Results

### Demographics and etiologies

**Figure 1:**
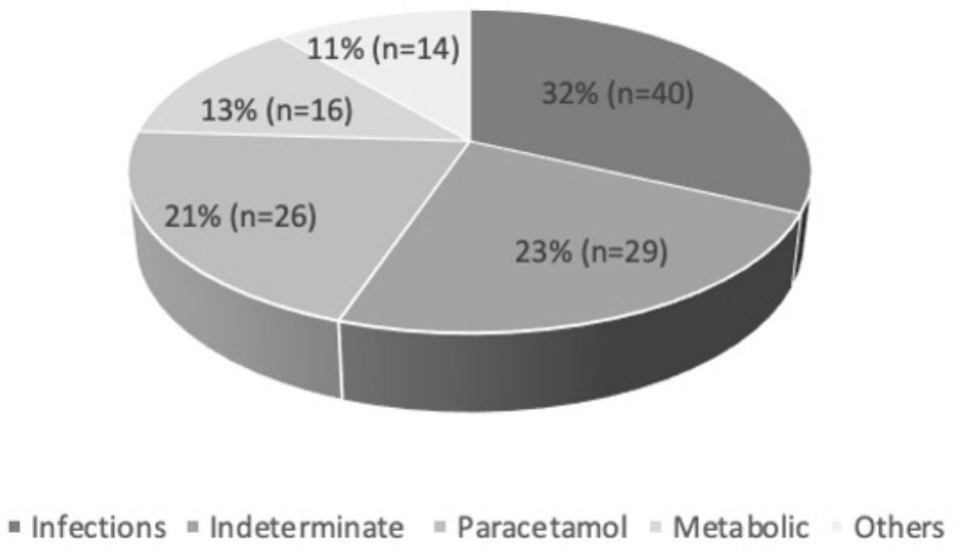
Etiologies of pediatric acute liver failure.

A total of 125 PALF patients were enrolled. Table 1 summarizes the demographic features of the study cohort. Out of 125 patients, 58.4% (*n* = 73) were male. PALF was more common in the 1–5 years age group 38% (*n* = 47) as compared to other age groups. The median age for SR and NSR group was 19 months (2 months−16 years) and 39.5 months (2 months−17 years), respectively (*p* = 0.010). The mean pediatric logistic organ dysfunction (PELOD) score for SR and NSR group was 9.58 and 17.13, respectively (*p* < 0.001). The mean length of stay (LOS) for SR and NSR was 4.63 days and 4.96 days, respectively (*p* = 0.73). The common causes of PALF (Figure 1.) were infections 32% (*n* = 40), followed by indeterminate 23% (*n* = 29), paracetamol toxicity 21% (*n* = 26), metabolic 13% (*n* = 16) and others 11% (*n* = 14). Dengue fever was the commonest infectious cause of PALF, accounting 55% of the patients (*n* = 22). Other infections causing PALF included sepsis induced multi-organ dysfunction syndrome (MODS) caused by Streptococcus pneumoniae, Staphylococcus aureus, Klebsiella pneumoniae, Acinetobacter species and Influenza (H1N1). Among 16 patients with metabolic causes resulting in ALF, four had Wilson disease. Apart from paracetamol toxicity, other drugs causing PALF included anti-tubercular drugs (*n* = 2), antiretroviral therapy (*n* = 1) and ayurvedic medicines [Indian native medicine] (*n* = 1). All patients who were admitted to the unit with ALF fulfilling KCHC or INR persistently > 4 for more than 24 h of PICU admission were given the option of referral to a transplant center.

**Table 1:** Patient demographics.

**Variables**	**Spontaneous** **regeneration** **(*n* = 79)**	**LT/Death** **(*n* = 46) ONLY 2** **PATIENTS HAD LT**	***P* value**
	***N* (%)**		
		***N* (%)**	
Gender			
Male	41 (51.9)	32 (69.6)	0.053
Female	38 (48.1)	14 (30.4)	
Jaundice	26 (32.9)	15 (32.6)	0.972
Hepatomegaly	74 (93.7)	42 (91.3)	0.622
Splenomegaly	9 (11.4)	7 (15.2)	0.537
Fever	63 (79.7)	32 (69.5)	0.199
Edema	44 (55.7)	31 (67.4)	0.198
Ascites	45 (56.9)	23 (50)	0.451
HE	51 (64.5)	34 (73.9)	0.28
PT >100 s	11 (13.9)	12 (26.1)	0.091
Bleeding	47 (59.5)	41 (89.1)	
Major	32 (40.5)	34 (73.9)	<0.001*
Minor	15 (18.9)	7 (15.2)	0.001*
MODS	19 (24)	36 (78.3)	<0.001*
Inotropes	25 (31.6)	43 (93.5)	<0.001*
NAC	32 (40.5)	9 (19.6)	0.016*
Mechanical ventilation	36 (45.5)	42 (91.3)	<0.001*
RRT	19 (24)	17 (36.9)	0.114
KCHC	22 (27.8)	16 (34.8)	0.416
INR > 4	36 (45.6)	32 (69.6)	0.009*

HE, Hepatic encephalopathy; INR, International normalized ratio; PT, Prothrombin time; MODS, Multi-organ dysfunction syndrome; NAC, N-acetyl cysteine; RRT, Renal replacement therapy; KCHC, King's College Hospital Criteria; Non-PCM, Non-paracetamol induced.

### Clinical manifestations

**Figure 2:**
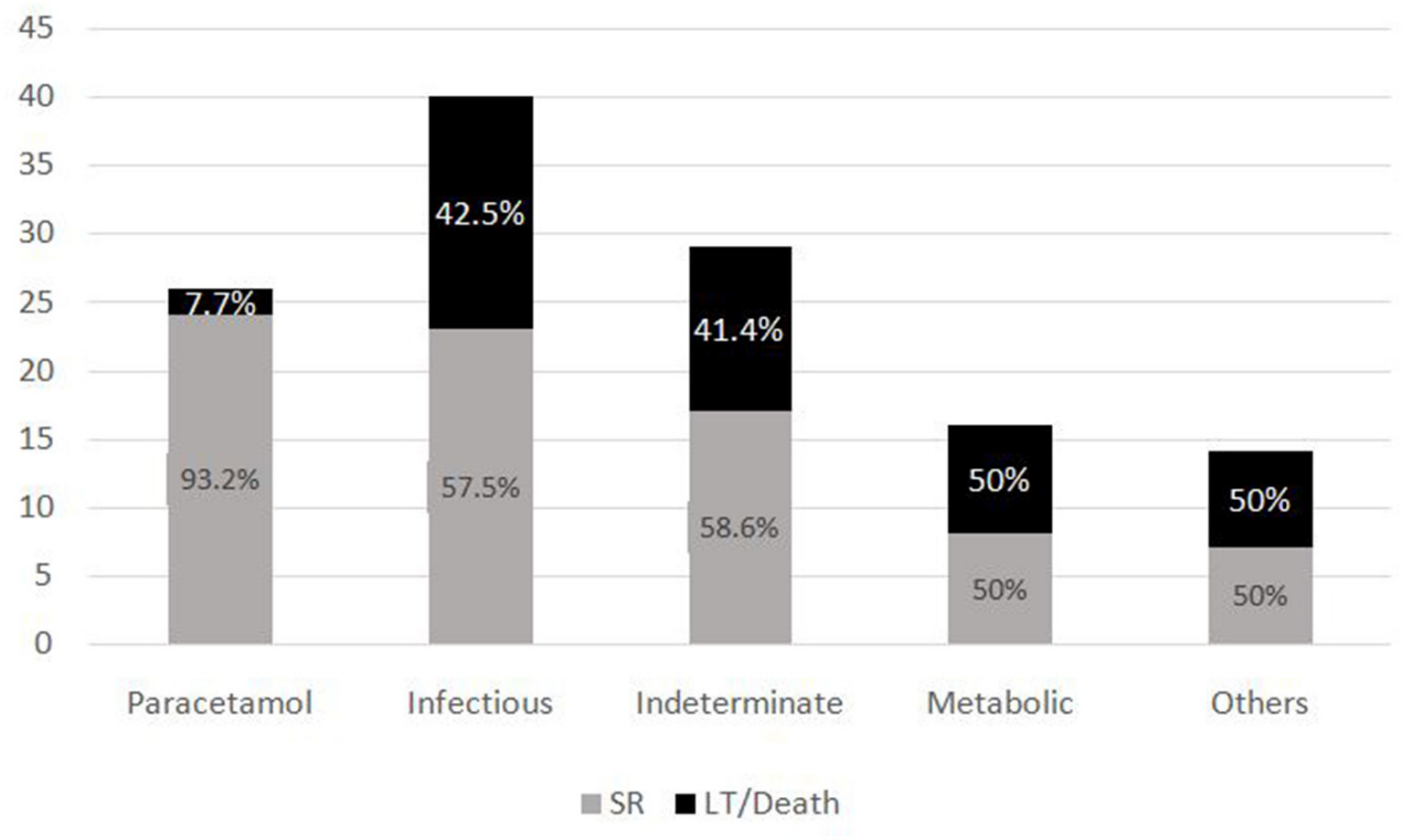
Outcome of patients based on etiologies.

**Figure 3:**
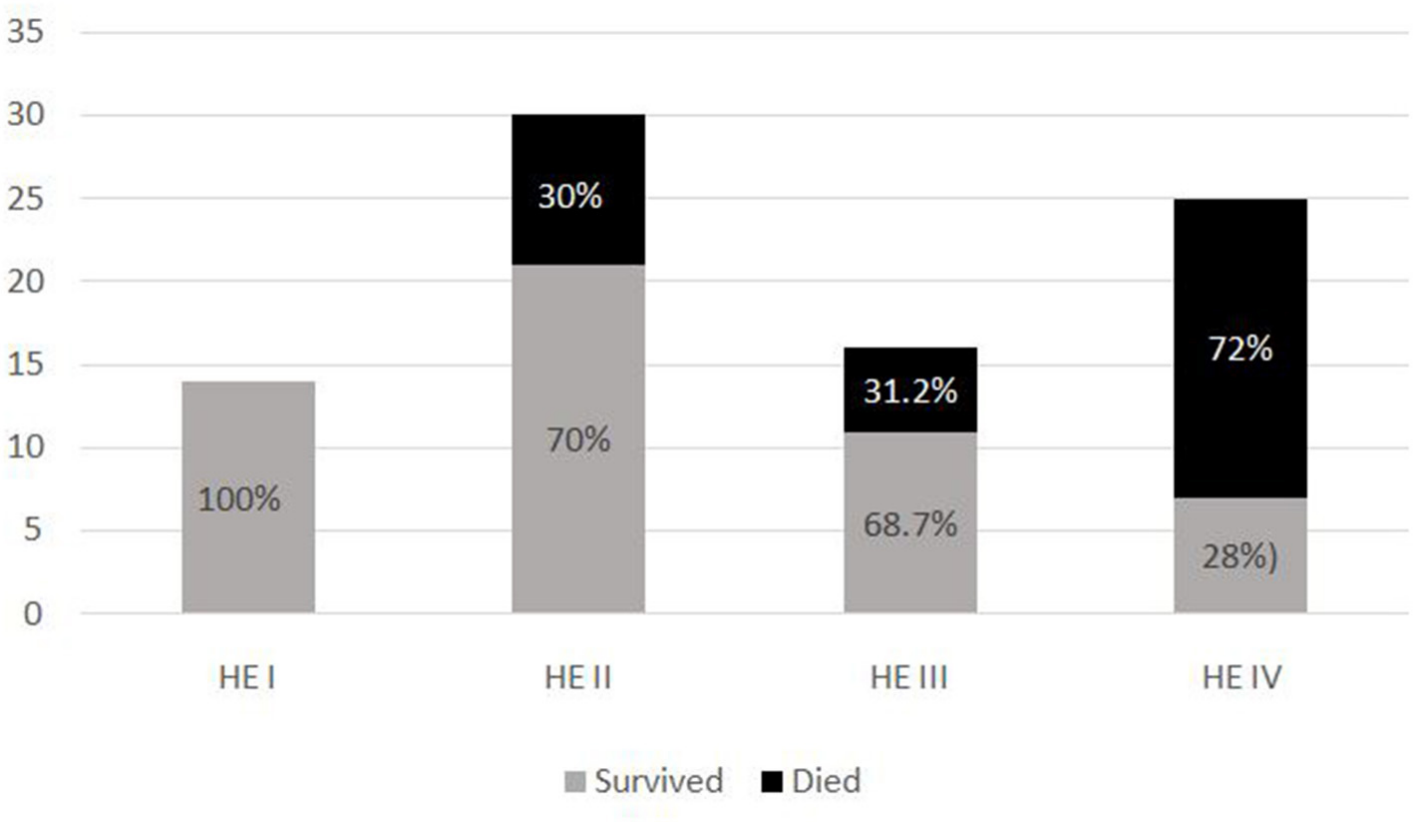
Outcome of patients based on grades of hepatic encephalopathy.

The most common symptom was bleeding, present in 70% (*n* = 88) of PALF patients. Among patients with bleeding, 75% had major bleeding. Gastrointestinal bleeding was present in 65.9% (*n* = 58) with skin and mucosal bleeding in 14.8% (*n* = 13), pulmonary bleeding was present in 5.6% (*n* = 5), and intracranial bleeding in 3.4% (*n* = 3). The most common sign was hepatomegaly, present in 92.8% (*n* = 116) patients. HE was present in 68% (*n* = 85) patients. Among these, 51.8 % (*n* = 44) had grade I-II HE and 48.2% (*n* = 41) patients had grade III–IV HE. Jaundice was present in 32% (*n* = 41) of patients. PALF was a part of MODS in 44% (*n* = 55) of patients. Amongst the 125 children with PALF, 30.4% (*n* = 38) patients satisfied KCHC and 59.2% (*n* = 74) fulfilled the criteria of INR > 4 for LT. Of the entire cohort, only two children who both had PALF secondary to Wilson disease, underwent LT and both survived. The clinical features and laboratory investigations of these groups are shown in Tables 1, 2. SR occurred in 63.2% (*n* = 79). The rate of SR was better in patients with PCM induced liver failure [92.3% (*n* = 24/26)], compared to non-PCM induced liver failure [55.5% (*n* = 55/99)] (Figure 2). Of 44 patients with HE grade I-II, 77.3% (*n* = 34) had SR, whereas of the 41 patients with grade III-IV HE, only 41.5% (*n* = 17) patients had SR (*p* = 0.001). The presence of HE grade III-IV was associated with poor outcome (Figure 3.). The patients who were in the NSR group were sicker than the ones in the SR group and had significantly higher incidence of bleeding, inotropic requirement, mechanical ventilation and MODS, as shown in Table 1. Biochemically, children in the NSR group had higher peak ammonia, peak lactate and direct bilirubin when compared to SR group, as shown in Table 2.

**Table 2:** Laboratory investigations.

**Variables**	**Spontaneous** **Liver** **regeneration** **(*n* = 79)** **^a^Median (Min/Max)/ ^b^Mean (SD)**	**LT/Death** **(*n* = 46)** **Median (Min/Max)/** **Mean (SD)**	***P*-value**
^a^Serum bilirubin (mg/dl)	1.6 (0.3/30.2)	3.2 (0.3/38.7)	0.062
^a^Admission PELOD Score	9 (4–19)	17 (5–27)	<0.000
^a^Direct bilirubin (mg/dl)	0.9 (0.1/23.2)	1.9 (0.1/30.4)	0.035*
^a^Peak lactate (mmol/l)	4.1 (1/25)	9.4 (2/32)	<0.001*
^a^Peak creatinine (mg/dl)	0.52 (0/5)	0.57 (0.1/5.2)	0.191
^a^Peak ammonia (μmol/L)	96 (10/392)	140 (41/967)	0.025*
^a^Lowest pH	7.3 (6.7/7.5)	7.13 (6.6/7.4)	<0.001*
^b^Peak aspartate transaminase (IU/L)	5,623.87 (6,104.94)	3,594.39 (4,787.02)	0.055
^b^Peak alanine transaminase (IU/L)	3,658.65 (3,883.42)	1,855.30 (3,037.01)	0.008*
^b^Lowest albumin (mg/dl)	2.75 (0.62)	2.35 (0.62)	0.001*
^b^Baseline creatinine (mg/dl)	0.43 (0.39)	0.56 (0.65)	0.191
^b^Lowest sodium	133.39 (4.46)	132.63 (6.74)	0.449
^b^Prothrombin time (s)	48.45 (25.24)	55.44 (23.60)	0.165
^b^INR	4.10 (2.23)	4.95 (2.22)	0.063
^b^Serum PCM level (35/4)	42.09 (29.45)	38.1 (24.72)	0.797

^a^Median (Minimum/Maximum); ^b^Mean ± SD.

LOS, Length of stay; INR, International normalized ratio; PELOD, Pediatric Logistic Organ Dysfunction; PCM, Paracetamol.

### Interventions

During the PICU stay, 54.4% needed inotropes, 62.4% were invasively ventilated, and 28.8% patients underwent RRT. In our cohort, 32.8% (*n* = 41) received N-acetyl cysteine (NAC) during the PICU stay, of which 63.4% (*n* = 26) were in the PCM toxicity group. Among the 36 patients requiring RRT, 50% (*n* = 18), 27.8% (*n* = 10), and 22.2% (*n* = 8) underwent intermittent hemodialysis, peritoneal dialysis and sustained low-efficiency dialysis, respectively. During the study period, continuous renal replacement therapy was not available at our center. In this cohort, for all the children meeting KCHC or INR > 4 criteria, parents were counseled and advised for requirement of LT, of which only 16 parents opted for LT due to financial constraints and were subsequently referred for LT to other centers on parents' agreement. Amongst these 16 patients, 56.2% (*n* = 9) patients fulfilled both KCHC and INR > 4 criteria, 25% (*n* = 4) patients fulfilled INR > 4 criteria and 18.7% (*n* = 3) fulfilled KCHC. Of these 16 patients, 50% (*n* = 8) patients survived. Of eight patients who survived, two children underwent LT and 6 survived without LT. All 8 patients who died were on the waiting list for LT.

### Outcome

On univariate analysis, age, use of inotropes, requirement of mechanical ventilation, presence of bleeding, MODS, HE grade >III, lowest pH, peak lactate, peak alanine transferase (ALT), peak ammonia and PELOD score significantly influenced the outcome of patients - death/LT (Tables 1, 2). On multivariate logistic regression analysis, only ALT and use of inotropes were found to be significantly associated with outcome of patients. The mortality rate was the lowest (7.7%) in the group with PCM induced ALF. Among the survivors, only five patients had abnormal neurological exam at the time of discharge of which only one patient had severe disability with hypoxic ischemic encephalopathy changes, while the rest had minor abnormalities or findings related to critical neuromuscular illness.

#### Validity of LT criteria for predicting death without liver transplantation for PALF-

We tested the KCHC as well as UK organ allocation criteria of INR > 4 to see if these 2 criteria predicted survival with SR or death/LT.

#### Validity of KCHC in entire cohort

In this cohort, 30.4% (*n* = 38) met the KCHC. Of these 38 children, 57.9% (*n* = 22) had SR, 5.3% (*n* = 2) survived after LT, and 36.8% (*n* = 14) died. Similarly, 69.6% (*n* = 87) patients did not meet the KCHC, of whom 65.5% (*n* = 57) had SR and 34.5% (*n* = 30) did not.

#### Validity of KCHC in non-PCM induced PALF group

Of the 99 patients in the non-PCM induced ALF group, 26.3% (*n* = 26) met the KCHC for LT. Of these, 26 children, 42.3% (*n* = 11) had SR, 7.7% (*n* = 2) survived after LT, and 50% (*n* = 13) died. Of the 73 patients who did not meet KCHC, 60.3% (*n* = 44) had SR and 39.7% (*n* = 29) did not.

#### Validity of INR > 4 criterion

In our cohort of patients, 59.2% (*n* = 74) met the criterion of INR>4 for LT. Of these 54% (*n* = 40) had SR, 2.7% (*n* = 2) survived after LT, and 43.2% (*n* = 32) died. Similarly, 40.8% (*n* = 51) who did not met INR > 4 criteria for LT, 76.4% (*n* = 39) had SR whereas 23.5% (*n* = 12) did not.

SR was not significantly different among those who did and did not meet KCHC, for the whole cohort (*p* = 0.416) as well as for subgroup with non-PCM induced PALF (*p* = 0.113). The UK organ allocation criteria for LT based on INR > 4 was better in predicting outcomes of PALF (*p* = 0.011). The sensitivity, specificity, positive and negative predictive values of KCHC for whole cohort and non-PCM induced PALF and INR > 4 are shown in Table 3. Despite fulfilling the criteria of KCHC (30.4%) and INR > 4 (59.2%) for LT, only 2 patients underwent LT.

**Table 3:** Validity of liver transplantation criteria.

	**KCHC**	**KCHC (Non-PCM)**	**INR > 4**
Sensitivity (%)	34.78	31.7	69.57
Specificity (%)	72.15	80.3	54.43
PPV (%)	42.11	54.2	47.1
NPV (%)	65.5	61.6	75.44

PPV, Positive Predictive value; NPV, Negative Predictive value.

The optimal cut–off points for predicting poor outcome of PALF for total serum bilirubin was 2.55 mg/dl [Area–under–curve (AUC) 0.64; 95% confidence interval 0.54–0.74], ALT was 990 IU/L [AUC 0.31; 95% CI 0.21–0.41], ammonia was 120.50 (μmol/L [AUC 0.66; 95% CI 0.55–0.77], INR was 4.35 [AUC 0.639; 95% CI 0.541–0.737], lactate was 4.95 mmol/l [AUC 0.73; 95% CI 0.64–0.82], pH was 7.22 [AUC 0.24; 95% CI 0.16–0.33], albumin was 2.35 [AUC 0.33; 95% CI 0.23–0.42], lowest sodium was 132.5 mmol/l [AUC 0.46; 95% CI 0.35–0.57], and PELOD was 12.5 [AUC 0.88; 95% CI 0.82–0.94], and LIU score was 226.6 [AUC 0.59; 95% CI 0.47–0.72]. Refer to Supplemental materials.

## Discussion

Despite the advances made in the diagnosis and management of PALF, ideal prognostic criteria to predict outcomes of children with ALF are still lacking. Although LT is the only definitive treatment for PALF for patients who are thought to be unlikely to recover with supportive and intensive care management, however there are increasing reports of SR of patients with ALF (18). However, there continue to be reports of poor outcome of children with ALF in resource limiting settings where financial constraints and limited availability of donor organs are the main challenges. LT is an expensive and risky procedure, with post-transplant mortality rates as high as 25.5% (20). In addition, there is significant morbidity and a small risk of mortality to the donor. Therefore, there is a need to better understand the current outcomes of PALF and develop better prognostic criteria, especially for PALF in resource limited settings. The derivation of the clinical and laboratory prognostic factors for PALF would help us to predict which children would spontaneously recover and who would require LT. This will allow continuation of medical management in patients who are predicted to recover spontaneously, thereby avoiding unnecessary LT and consideration of LT for those patients who are unlikely to recover with supportive management alone. Most of the literature in PALF is reported from high income countries (HICs) where LT is more easily accessible, whereas this study was done in a resource limited setting to identify the etiologies of PALF, prognostic factors to predict the outcome of PALF as well as the validity of LT criteria (KCHC and INR > 4) for LT referral.

The indeterminate etiology accounts for more than 50% for PALF in all age groups in the United Kingdom (6, 9, 12, 13, 21), which is in contrast to our study where the commonest etiology of PALF was infection (32%). The reason for this may be due to the higher prevalence of infections leading to PALF in our setting. The indeterminate etiology has poor prognosis (18, 22) which may explain the findings in our study regarding increase in SR, since there were fewer patients with indeterminate etiology in this cohort. Studies by Figen et al. (23) and Zeren et al. (24) reported hepatitis A as the most frequent infectious cause of PALF in 76 and 66.7%,respectively. Similarly, studies from India published between 1996 and 2007, enrolling 215 children also showed acute viral hepatitis to be the commonest cause of PALF, either alone or in combination (25–31). These findings are in contrast to our findings where the dengue virus was the commonest infectious cause (55%) of PALF. The reason for these differences may be due to wider coverage vaccination for viral hepatitis, improved sanitation and the increasing prevalence of dengue fever in this territory.

In our study the most common clinical presentation of ALF was bleeding (70%) with gastrointestinal bleeding accounting for 94% of cases. This rate of bleeding seems to be much higher than what has been reported in the western cohorts (32), where the overall incidence of bleeding was only 10.6%. In our cohort of patients, dengue fever was the commonest infectious cause of PALF, accounting 55% of the patients (*n* = 22). Gastrointestinal bleeding is one of the commonest sites of bleeding in dengue (e.g., gum bleeding, melena) as these patients have thrombocytopenia, endothelial dysfunction and coagulopathy whereas patients with PALF due to other etiologies of PALF have relatively less bleeding due to a prothrombotic effect. Hence, variation in etiologies of PALF in different geographic locations of various countries could have been contributed to increase in the rate of bleeding in our study.

Previous studies have reported the rates of SR in ALF patients to be between 56% (12) and up to 73.4% (13). This also holds true in our cohort of patients as, where 63.2% of children with PALF had SR. This may be due to several factors including (1) early diagnosis (2) different etiologies, especially infectious 3) better understanding and advancement of medical care.

The subgroup analysis of the PALF Study database (6) demonstrates a 94% SR of PCM-induced PALF, similar to our study where we found that 92.3% of PCM-induced toxicity had SR. Several studies (6, 9, 13) have described about various etiologies of PALF being related to prognosis of disease in children. These studies have showed that there is increased chance of SR in conditions like PCM toxicity, hepatitis A and ischemic hepatitis, whereas the chances of SR are lower in indeterminate and metabolic causes. This is in contrast to our findings, wherein 58.6% of patients with indeterminate and 50% of metabolic category still had spontaneous regeneration. However, when compared to PCM toxicity, SR was lower in these groups. Hence, we may need to make etiology specific prognostic models for PALF in resource limited settings.

The KCHC were formulated in 1989. The original derivation cohort for this model included 588 patients (both children and adults) in the pre-LT era. The positive predictive value (PPV) for mortality in non-paracetamol induced ALF was 97%, indicating a high risk of death if meeting the criteria (15). Subsequent studies, primarily performed in adults, have demonstrated similar findings in non-PCM induced ALF, with PPV ranging from 80–96% and negative predictive value (NPV) ranging from 42–82% (33–39). These findings are in contrast to our study where sensitivity (31.7%) and PPV (54.2%) of KCHC were low - i.e., the KCHC does not reliably predict that a patient with non-PCM induced ALF will die if criteria are met. Whereas it is more likely to predict SR if the criteria are not met, as it has high specificity (80.3%) and NPV (61.6%). This finding is similar to the study done by Sundaram et al. (40) where sensitivity (61%) and PPV (33%) of KCHC for mortality were lower while its specificity (70%) and negative predictive value was higher (88%). In our study, 61.1% of those who met KCHC still survived without LT, whereas of those who did not meet KCHC 34.5% still died. Hence these results raise some important questions about (1) validation of KCHC in resource limited countries where infectious etiologies predominate (2) need for development of prognostic criteria for PALF to predict SR and those who need LT in resource limited settings. In the original KCHC cohort of 588 patients, the mortality risk was approximately 80% if they fulfilled the criteria in the original KCHC cohort of 588 patients. Also, it has been mentioned and known that etiology of PALF may play a substantial role in the prognosis. Therefore, the KCHC may not perform well in our population where the etiologies are varied and different. Hence, it would be useful to search for better predictors which suits to our population and etiologies, and proceeding to transplantation based on these factors.

There was higher mortality in our cohort (35.2%) compared to other western cohorts (16.4%) (6). One reason for this could be that our cohort of children were quite seriously ill at the time of presentation [Median PELOD score-17 (5–27)]. Other reasons could be delay in seeking health care services in resource limited settings due to financial constraints and challenges to accessibility to health care services. At this point we are not sure whether our cohort could have benefitted from LT even if it were available, because despite being sicker (54.4% needed inotropes and 62.4% were invasively ventilated), SR was seen in 63.2% of patients. This again could be attributed to the underlying etiology leading to ALF.

Various previous studies have studied several clinical and laboratory prognostic factors for PALF. A study from King's College Hospital (11) in the pre-transplantation era showed that there is a higher chance of death if PT > 90 seconds. Subsequent studies done after LT revealed that PT and INR were significant predictors of death/LT or death alone (13, 41, 42). These findings are similar to our results. There are different thresholds of INR values mentioned in the literature. According to the PALF Study database INR > 2.5 is a significant predictor of death whereas in the UK organ allocation is based on INR > 4 (18). This was similar to our findings as INR > 4 was statistically significant to predict outcome in our cohort of patients (*p* = 0.01).

In our study the severity of HE was significantly associated with outcome of patients with PALF. Of 41 patients with grade III-IV HE, 52.2 % did not recover spontaneously or required LT. Various studies (6, 11, 37, 41) have showed similar results, with the severity of HE being one of the poor prognostic factors of PALF.

The Liver Injury Units Scoring System (LIU) has been used for predicting outcome of patients in PALF (42). This study revealed that c-index from ROC curves for LIU score predicted LT better [0.84; 95% CI 0.80–0.87] than it did for death without LT [0.76; 95% CI 0.70–0.80]. As, only 2 patients underwent LT, LIU score for predicting LT was not done in our study. The AUC of LIU score using peak INR for predicting death in our study cohort was 0.59 (95% CI 0.47–0.72) which was lower compared to the study by Brandy et al. which showed c-index of 0.76 (95% CI 0.70–0.82) (19). This difference may be due the difference in etiologies as well as small sample size in our cohort of patients.

Early initiation of CRRT has shown to favorably affect the outcome of children that leads to increase in survival as well as improves transplant-free survival in PALF (43). In our cohort 36 patient underwent RRT (Intermittent Hemodialysis, peritoneal dialysis and SLED), since CRRT was not available in our center during the study period. Non-availability of CRRT could have been a major confounding factor in determining outcome in our cohort.

The main limitation of this study was that this was a retrospective study confined to a single center, with a relatively small sample size. Due to the small sample size in individual etiologies, we were unable to do etiology specific prognostic modeling. It is difficult to extrapolate these findings due to different settings as etiologies are different and our patients had mainly infectious causes. Another limitation of this study is that we are unable to collect all the details of data of all patients who were transferred out from our hospital to a liver transplant center. Therefore, it was difficult to predict if KCHC or INR > 4 criteria had accurately predicted the need for transplant in these patients. Other limitations are unavailability of LT and CRRT facilities in our center. Hence, many of the children in our cohort might have benefitted from LT if it was available in our center.

## Conclusion

Infections are the commonest cause of PALF, with dengue fever being the predominant infectious cause, in our population. Children with paracetamol induced ALF had a better chance of spontaneous regeneration. Patients with high peak alanine aminotransferase, those requiring inotropes and advanced HE with high likelihood of death could be potentially considered for LT as available. The KCHC or INR >4 criteria alone may not be enough to predict outcome in PALF with different etiologies. Further multi-centric prospective studies are needed to determine better predictors of outcome of children with ALF in resource limited settings.

## Data availability statement

The original contributions presented in the study are included in the article/Supplementary materials, further inquiries can be directed to the corresponding author.

## Ethics statement

The studies involving human participants were reviewed and approved by the Kanchi Kamakoti CHILDS Trust hospital's Institutional Review Board (IRB) - approval number KKCTH-CTMRF: IEC-DNB/37/March 2019 (IRB min. dt. 07.03.2019). Written informed consent from the participants' legal guardian/next of kin was not required to participate in this study in accordance with the national legislation and the institutional requirements.

## Author contributions

PA: data collection, analysis, writing, reviewing, and revising the manuscript. SK: conception of idea, design of the study, analysis, and reviewing the manuscript. AD: critical reviewing of the manuscript. SS, RK, and KS: conception of idea and reviewing of manuscript. BR: conception of idea, reviewing and revision of the manuscript. All authors contributed to the manuscript revision and approved the submitted version.

## Conflict of interest

The authors declare that the research was conducted in the absence of any commercial or financial relationships that could be construed as a potential conflict of interest.

## Publisher's note

All claims expressed in this article are solely those of the authors and do not necessarily represent those of their affiliated organizations, or those of the publisher, the editors and the reviewers. Any product that may be evaluated in this article, or claim that may be made by its manufacturer, is not guaranteed or endorsed by the publisher.

## Abbreviations

SR: Spontaneous regeneration
NSR: Non-spontaneous regeneration.
