# Supplementary material for: Pediatric acute liver failure: An experience of a pediatric intensive care unit from resource limited settings
Source: Front Pediatr. 2022 Sep 2;10:956699. doi: 10.3389/fped.2022.956699 (PMC9478462; doi:10.3389/fped.2022.956699)

**Supplemental Table 1. Validity of King’s College Criteria for whole cohort**

| KCHC | Spontaneous recovery  N (%) | No spontaneous recovery N (%) | Total  N (%) | P value |
| --- | --- | --- | --- | --- |
| Yes | 22 (57.9) | 16 (42.1) | 38 (30.4) | 0.416 |
| No | 57 (65.5) | 30 (34.5) | 87 (69.6) |  |
| Total | 79 (63.2) | 46 (36.8) | 125 (100) |  |

**Supplemental Table 2. Validity of King’s College Criteria for Non-paracetamol-induced liver failure**

| KCHC | Spontaneous recovery  N (%) | No spontaneous recovery N (%) | Total  N (%) | P value |
| --- | --- | --- | --- | --- |
| Yes | 11 (42.3) | 15 (57.7) | 26 (26.3) | 0.113 |
| No | 44 (60.3) | 29 (39.7) | 73 (73.7) |  |
| Total | 55 (55.5) | 44 (44.4) | 99 (100) |  |

**Supplemental Table 3. Validity of INR>4 Criteria for whole cohort**

| INR >4 | Spontaneous recovery  N (%) | No spontaneous recovery N (%) | Total  N (%) | P value |
| --- | --- | --- | --- | --- |
| Yes | 40 (54.0) | 34 (45.9) | 74 (59.2) | 0.011* |
| No | 39 (76.4) | 12 (23.5) | 51 (40.8) |  |
| Total | 79 (63.2) | 46 (36.8) | 125 (100) |  |

**Supplemental Table 4. Cut-off values for predicting mortality of pediatric acute liver failure.**

| **Variables** | **Area-under-curve (AUC)** | **95% Confidence Interval (CI)** | **Cut-off values** |
| --- | --- | --- | --- |
| Total serum bilirubin | 0.639 | 0.537-0.741 | 2.55 |
| Alanine transaminase | 0.312 | 0.214-0.409 | 990 |
| Aspartate transaminase | 0.373 | 0.270-0.475 | 1807 |
| pH | 0.245 | 0.158-0.333 | 7.22 |
| Albumin | 0.328 | 0.231-0.425 | 2.35 |
| Ammonia | 0.662 | 0.551-0.774 | 120.50 |
| Lactate | 0.731 | 0.639-0.823 | 4.95 |
| Creatinine | 0.576 | 0.474-0.678 | 0.515 |
| PLEOD | 0.879 | 0.816-0.942 | 12.5 |
| Lowest sodium | 0.461 | 0.351-0.571 | 132.50 |
| INR | 0.639 | 0.541-0.737 | 4.35 |
| LIU score | 0.597 | 0.473-0.720 | 266.6 |

**Supplemental Figure 1.** **ROC for total serum bilirubin**


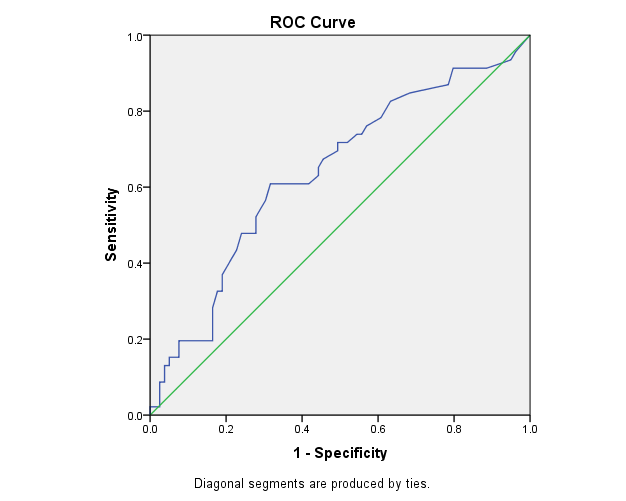


**Supplemental Figure 2.** **ROC for alanine transaminase**


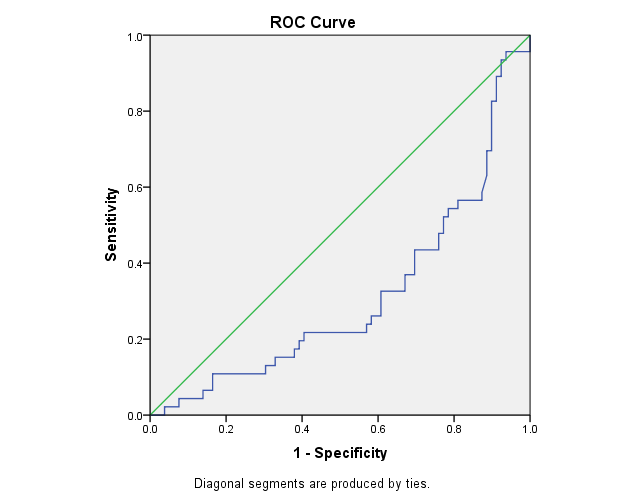


**Supplemental Figure 3.** **ROC for albumin**


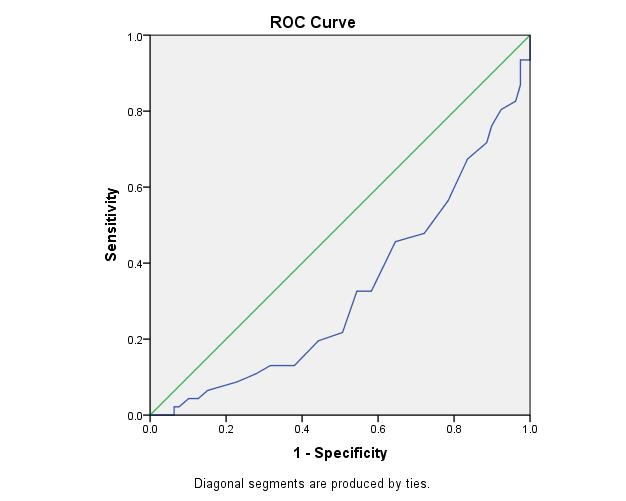


**Supplemental Figure 4.** **ROC for ammonia**


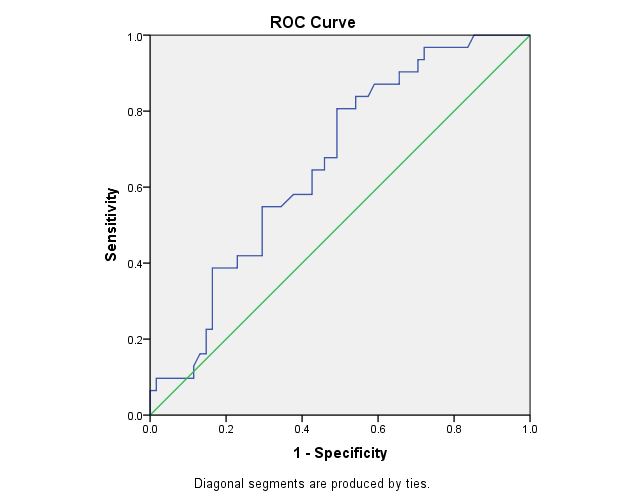


**Supplemental Figure 5.** **ROC for lactate**


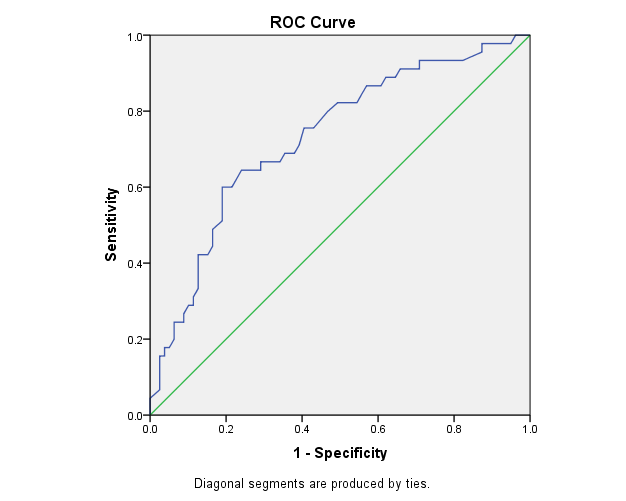


**Supplemental Figure 6.** **ROC for pH**


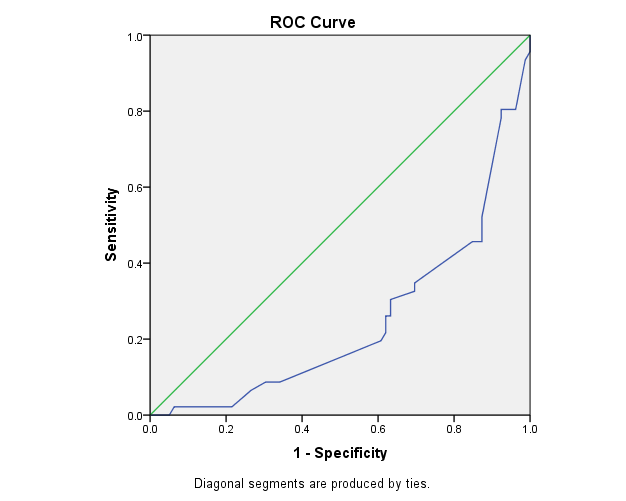


**Supplemental Figure 7.** **ROC for PELOD**


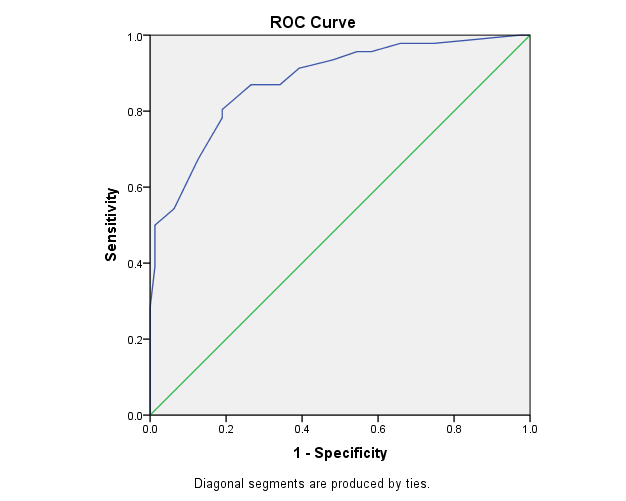


**Supplemental Figure 8.** **ROC for lowest sodium**


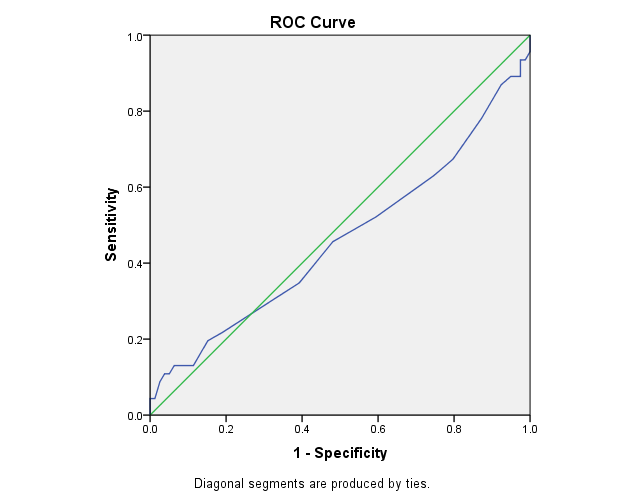


**Supplemental Figure 9.** **ROC for INR**


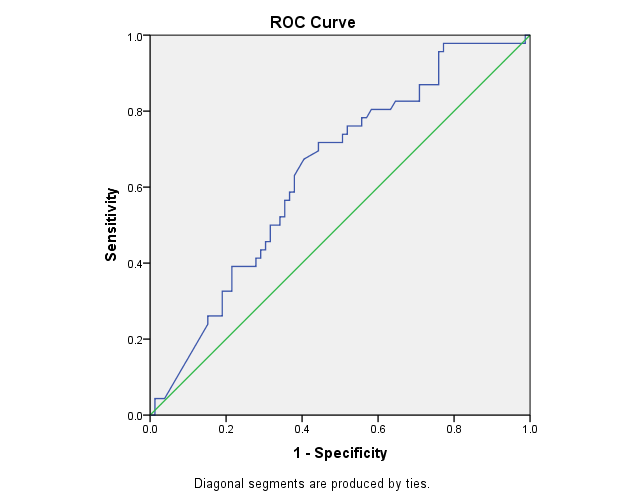


Supplemental Figure 10. ROC for Liver Injury Units (LIU) score


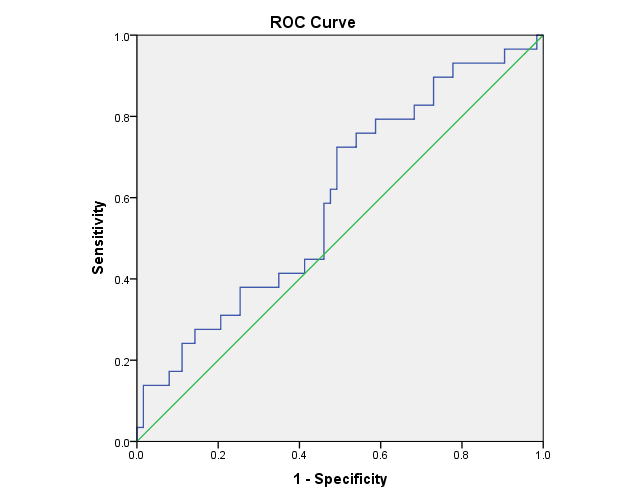

Supplement: Supplementary file 1 [file Data_Sheet_1.docx]
